# Nutritional Quality of Pasta Sold on the Italian Market: The Food Labelling of Italian Products (FLIP) Study

**DOI:** 10.3390/nu13010171

**Published:** 2021-01-08

**Authors:** Marika Dello Russo, Carmela Spagnuolo, Stefania Moccia, Donato Angelino, Nicoletta Pellegrini, Daniela Martini, on behalf of the Italian Society of Human Nutrition (SINU) Young Working Group

**Affiliations:** 1 Institute of Food Sciences, National Research Council, 83100 Avellino, Italy; marika.dellorusso@isa.cnr.it (M.D.R.); carmela.spagnuolo@isa.cnr.it (C.S.); stefania.moccia@isa.cnr.it (S.M.); 2 Faculty of Bioscience and Technology for Food, Agriculture and Environment, University of Teramo, 64100 Teramo, Italy; dangelino@unite.it; 3 Department of Agricultural, Food, Environmental and Animal Sciences, University of Udine, 33100 Udine, Italy; 4 Department of Food, Environmental and Nutritional Sciences (DeFENS), Università degli Studi di Milano, 20133 Milan, Italy; daniela.martini@unimi.it

**Keywords:** pasta, food labelling, nutrition declaration, nutritional composition, gluten free, nutrition claims

## Abstract

Pasta represents a staple food in many populations and, in recent years, an increasing number of pasta items has been placed on the market to satisfy needs and trends. The aims of this work were: (i) to investigate the nutritional composition of the different types of pasta currently sold in Italy by collecting the nutrition facts on their packaging; (ii) to compare energy, nutrient and salt content per 100 g and serving in fresh and dried pasta; (iii) to compare the nutrition declaration in pairs of products with and without different declarations (i.e., gluten free (GF), organic, and nutrition claims (NC)). A total of 756 items, made available by 13 retailers present on the Italian market, were included in the analysis. Data showed a wide difference between dried and fresh pasta, with high inter-type variability. A negligible amount of salt was observed in all types of pasta, except for stuffed products, which had a median high quantity of salt (>1 g/100 g and ~1.5 g/serving). Organic pasta had higher fibre and lower protein contents compared to conventional pasta. GF products were higher in carbohydrate and fat but lower in fibre and protein than not-GF products, while only a higher fibre content was found in pasta with NC compared to products not boasting claims. Overall, the results show high variability in terms of nutrition composition among the pasta items currently on the market, supporting the importance of reading and understanding food labels for making informed food choices.

## 1. Introduction

Pasta is one of the most widespread staple foods, known at least since the time of the Etruscans, who learned how to work the wheat by grinding it, mixing it with water, levelling it in thin doughs, and cooking it on a red-hot stone. According to Italian law [1], “dried pasta” must be made with water and durum wheat semolina, while “fresh pasta” can be made with soft wheat and has a higher moisture content than “dried pasta”. There are also laws regarding the preparation of “special pasta”, which contains other ingredients than wheat and water: “egg pasta”, manufactured with durum wheat and hen’s eggs, and “stuffed pasta”, which includes “fresh pasta” filled with different ingredients, as in the case of ravioli or lasagne. In this scenario, the manufacturing process of pasta is continuously updated over the years to face food needs and trends. For example, a wide range of pasta containing different ingredients, such as vegetable extracts, i.e., spinach and tomato, is currently available on the market.

A survey carried out by the International Pasta Organisation in 2014 reported that about 14.3 million tons of pasta are annually produced worldwide, mainly in Italy, the United States, Brazil, Turkey and Russia. Italians are the main pasta consumers, with 25.3 kg per capita per year, followed by Tunisians (16 kg), Venezuelans (12.2 kg) and Greeks (11.5 kg) [2]. Pasta consumption has faced a decrease in the last years, probably because of, among other reasons, the myth about dodging carbohydrate-rich foods as a “strategy” for losing weight [3]. However, it is worth noting that pasta is a key component of many healthy eating patterns, above all the Mediterranean Diet, and its consumption has been positively associated with a low body mass index and prevention of overweight and obesity risk conditions [4,5,6,7,8]. Among the possible reasons explaining the positive effects it has on health, pasta generally has a good nutritional quality, due to low amounts of fat and available carbohydrates. Moreover, pasta can be also a suitable vehicle for the incorporation of beneficial components, such as fibre or probiotics [9,10], considering its low cost, long shelf life, and wide range of acceptability in many consumers groups [11]. However, pasta is a very heterogeneous category, including several types of products which often differ not only in shape but most importantly in ingredients, and thus, in nutrition composition. Nevertheless, dietary recommendations do not take into account this variability for suggestions in portion sizes and frequency of consumption. Moreover, it should be carefully considered that pasta is often consumed in association with other ingredients, i.e., oil and/or grated cheese, or elaborated sauces, which can largely contribute to the energy value of the entire serving. Regardless of this aspect, the knowledge of the nutritional quality of pasta itself can be useful for evaluating the nutritional characteristics of the dish and helping consumers in their purchase. However, an overview of the nutritional quality of all the products named as “pasta” on the label is still missing in the literature.

In Europe, the nutrition information of pasta, and generally of pre-packed foods, is available to the consumer on the food label in accordance with Council Regulation No 1169/2011 [12], together with other mandatory information (i.e., list of ingredients, net amount, and name of producer). Moreover, other voluntary information can be reported on the pack, including nutrition claims (NC) and health claims (HC), the reduced presence or absence of gluten, or organic certification [12,13,14]. Research has shown that reading and understanding the nutrition facts and the claims reported on the label can help consumers in making healthy food choices [15]. However, it has been evidenced that the presence of NC or HC or the absence of gluten on the label can be misperceived as a guarantee of a better nutritional quality of the product [16,17,18,19]. Thus, consumers should be guided towards more informed and conscious food choices, which may lead to better dietary behaviours. In this context, the Food Labelling of Italian Products (FLIP) Study was conceived to systematically investigate the overall quality of the pre-packed foods of the most important food groups sold on the Italian market by collecting the nutrition declaration on their packaging [20,21]. The present study specifically focuses on pasta: there is, indeed, a wide variety of different pasta products sold on the shelves, and many of them boast nutrition claims and other information. A comparison of energy, nutrient, and salt content in pairs of products with and without the different declarations considered in the study (i.e., gluten free (GF), organic and their counterparts, as well as NC) was performed. Moreover, even if pasta is commonly consumed accompanied by other ingredients and sauces, the nutritional characteristics of pasta itself might be different among the various types, which may influence the nutritional quality of the entire meal. Based on these premises, it is important to investigate the nutritional composition of pasta sold in the market, also considering that the serving size can be widely different among the different types of pasta. To the best of our knowledge, no study has been carried out yet to comprehensively and systematically investigate the nutritional composition of the different varieties of pasta sold in Italy.

## 2. Materials and Methods

### 2.1. Food Product Selection

In the present study, information about pasta products was taken from the online shopping website of the main retailers present on the Italian market, as reported in a previous paper [21]. The online research was performed from July 2018 until March 2019.

All the prepacked pasta items with mandatory food information on the package, as requested by the Council Regulation (EC) no. 1169/2011 [12], were included. Conversely, the following products were excluded: not pre-packed, not available online during the collection data phase, with partial package images and/or unclear nutrition declaration, and/or an incomplete list of the ingredients. Pasta items were divided in two categories (fresh and dry), and for both, four types of pasta were selected and analysed: semolina, egg, stuffed, and special pasta.

### 2.2. Data Extraction and Analysis

For all the selected products, data from the complete images of the package were collected. The quali-quantitative data reported on the label of all products were recorded, including: company name, brand name, descriptive name, energy (kcal/100 g), total fat (g/100 g), saturated fatty acids (SFA, g/100 g), total carbohydrates (g/100 g), sugars (g/100 g), protein (g/100 g), and salt (g/100 g). In addition to the mandatory nutrition information indicated in the Council Regulation (EC) 1169/2011 [12], fibre content (g/100 g) was also collected. Descriptive name was used to classify the retrieved items in the two categories (fresh and dried pasta), each one including four types (semolina, egg, special, and stuffed pasta).

Once these values were retrieved, data of the energy and nutrient contents were also presented per standard serving by using the Italian suggested serving sizes for pasta [22]. The considered standard serving was 80 g for all types of dried pasta, 100 g for fresh semolina and egg pasta, and 125 g for fresh stuffed pasta.

Finally, information on the presence or absence of organic certification, GF declaration, and NC, was collected. Taking into account the disparity in the number of products with and without organic, GF, and NC declarations, the comparison of energy, nutrient, and salt content per 100 g in products with and without declarations was performed on only pairs of products, with three independent selections for each declaration. For each of the three declarations (organic, GF, NC), a similar item without declaration from the same brand was selected for each product by considering the category of pasta (i.e., fresh or dried) and type (i.e., semolina, egg, special, stuffed). For instance, for each fresh egg pasta GF, a corresponding fresh egg pasta item not GF from the same brand was chosen. When no items from the same brand but without the declaration were available, a similar item from the same category and type but another brand was randomly selected. Regarding GF products, the selection and the choice of pairs were limited to cereal-based products, thus excluding legume-based pasta, due to the high heterogeneity in terms of nutrition contents between these two types of products.

The precision of the extracted data was independently double-checked by two researchers (M.D.R. and S.M.), and inaccuracies were solved through secondary extractions by a third researcher (C.S.). After data collection, a dataset was created, grouping products into the four categories of interest: semolina pasta, egg pasta, stuffed pasta, and special pasta.

For the analysis of salt content, products were classified as “very low salt content” if they had <0.12 g of salt/100 g and “low salt content” if products had <0.3 g of salt/100 g, following the indications by Regulation (EC) No 1924/2006 [13]. The remaining products were instead classified as “medium salt content” (>0.3 but < 1 g of salt/100 g) and “high salt content” (≥ 1 g of salt/100 g) as reported by the Italian Society of Human Nutrition (www.sinu.it) in the dissemination materials produced for the “World salt awareness week” performed in the framework of the World Action on Salt and Health (http://www.worldactiononsalt.com/).

### 2.3. Statistical Analysis

The Statistical Package for Social Sciences software (IBM SPSS Statistics, Version 24.0, IBM corp., Chicago, IL) was used to perform the statistical analysis, with a significance level set at *p* < 0.05. The normality of data distribution was firstly verified through the Kolmogorov-Smirnov test and rejected. Therefore, variables were expressed as median and interquartile range. Differences in terms of energy, macronutrients, and salt content among different types of pasta were explored using the Kruskal-Wallis test for independent samples with multiple pairwise comparisons. Analysis per 100 g was performed separately within and not between the two pasta categories (fresh and dried pasta), due to the difference in their moisture content. The analysis per serving was instead performed among all different types of pasta. The Mann-Whitney non-parametric test for two independent samples was applied for the comparisons of organic, GF, or NC pasta with their relative counterparts.

## 3. Results

### 3.1. Number and Types of Products

A total of 756 different items were analysed, categorised in fresh pasta (*n* = 269) and dried pasta (*n* = 487) based on their legal name.

Moreover, according to the nutritional characteristics of the different types of pasta, products within each category were further grouped into four pasta types (i.e., semolina, egg, stuffed, and special pasta that include other ingredients, such as rice, quinoa, amaranth, and legumes). Among fresh pasta, stuffed pasta had the largest number of items (*n* = 208), followed by egg pasta (*n* = 45) and semolina pasta (*n* = 16), and only one special pasta item, which was, thus, not considered in the following analysis. Conversely, egg pasta prevailed among dried pasta (*n* = 206), followed by semolina (*n* = 157) and special pasta (*n* = 119), while only five stuffed pasta items were found.

To compare the nutritional composition between products with and without specific declarations, the analysis was carried out on 49 pairs of organic/conventional items, 90 GF/gluten-containing products. Finally, 45 products with at least one NC declaration and 45 without NC were considered.

### 3.2. Nutritional Composition of Pasta per 100 g

As reported in Table 1, there was a high variability of energy and nutrient contents among fresh and dried pasta (*p* < 0.05) when different pasta types, within each category, were analysed. Considering fresh pasta, egg pasta had total median energy of 293 (288–308) kcal/100 g, which was slightly but significantly higher than stuffed pasta, which had total median energy of 274 (248–293) kcal/100 g, and semolina pasta, which had total median energy of 272 (261–273) kcal/100 g. Different results were found for energy median values of dried pasta types, where no differences were observed between egg and stuffed pasta (369 (365–374) kcal/100 g and 394 (393–403) kcal/100 g, respectively), even though they had significantly higher energy values than special and semolina pasta (351 (347–358) kcal/100 g and 354 (351–357) kcal/100 g, respectively). Overall, carbohydrates were the most abundant macronutrients, ranging from 54% of energy in stuffed fresh pasta to 71% of energy in semolina dried pasta.

In the fresh pasta category, stuffed pasta showed a significantly higher content of total fat, SFA, sugar, and salt, but a significantly lower amount of carbohydrate, compared to the other pasta types. Among the dried pasta, semolina and special pasta had a significantly lower total fat, SFA, and protein content and a greater amount of carbohydrates compared to egg- and stuffed-pasta (Table 1).

**Figure 1:**
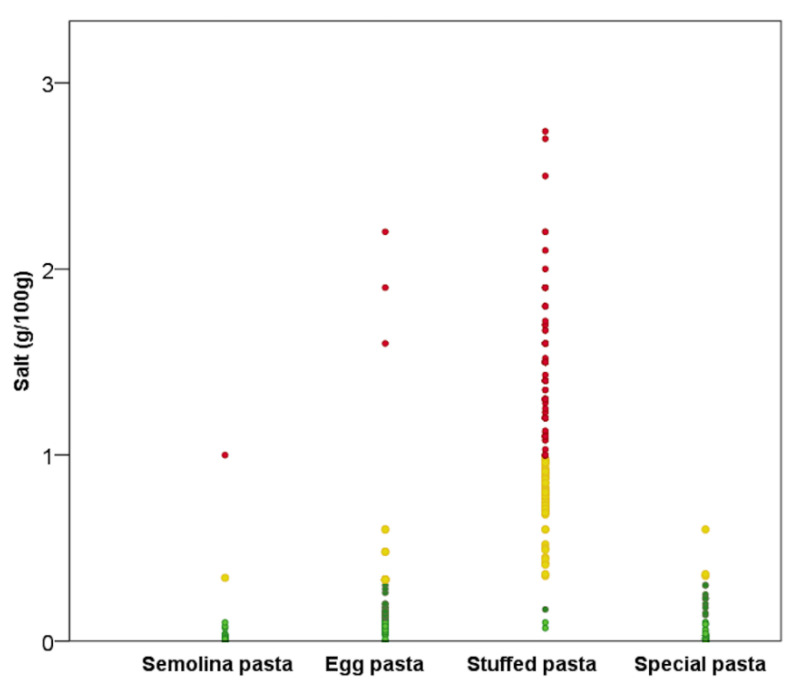
Salt content of the pasta products. Coloured dots refer to the classification for salt content by Council Regulation (EC) No 1924/2006 [13]: light green = very low salt content (<0.12 g/100 g); green = low salt content (<0.3 g/100 g); yellow = medium salt content (<1 g/100 g); red = high salt content (≥1 g/100 g).

Regarding salt content, only stuffed pasta, both fresh and dried, had a median high quantity of salt (>1 g/100 g). Considering all the items (i.e., fresh and dried), low content of salt (<0.3 g/100 g) was reported by 68.1% of the products, while a medium (>0.3 g/100 g but <1 g/100 g) and high (≥1 g/100 g) category of salt content was reported for 10.9% and 21.1%, respectively. As shown in Figure 1, the type of pasta with the highest salt content was stuffed pasta. Indeed, 98.6% of stuffed products had a medium or high salt content. Conversely, no special pasta items and only 0.6% of semolina products had a high salt content.

Table 2 shows the nutritional content of organic, GF, and NC pasta and their respective counterparts. No differences were observed when considering organic declaration, except for a lower protein content (12.0 (11.0–14.0) vs. 13.7 (13.0–14.6) g/100 g) and higher fibre content (3.0 (2.8–3.4) vs. 2.8 (2.7–3.0) g/100 g) in organic pasta compared to conventional pasta. GF products showed a significantly higher content of total carbohydrate and fat and a lower content of sugar, fibre and protein compared to the non-containing gluten counterpart (Table 2). No differences were identified when products with NC were compared to their counterpart, except for fibre content, which was significantly higher in pasta with NC.

### 3.3. Nutritional Composition per Serving Size

**Figure 2:**
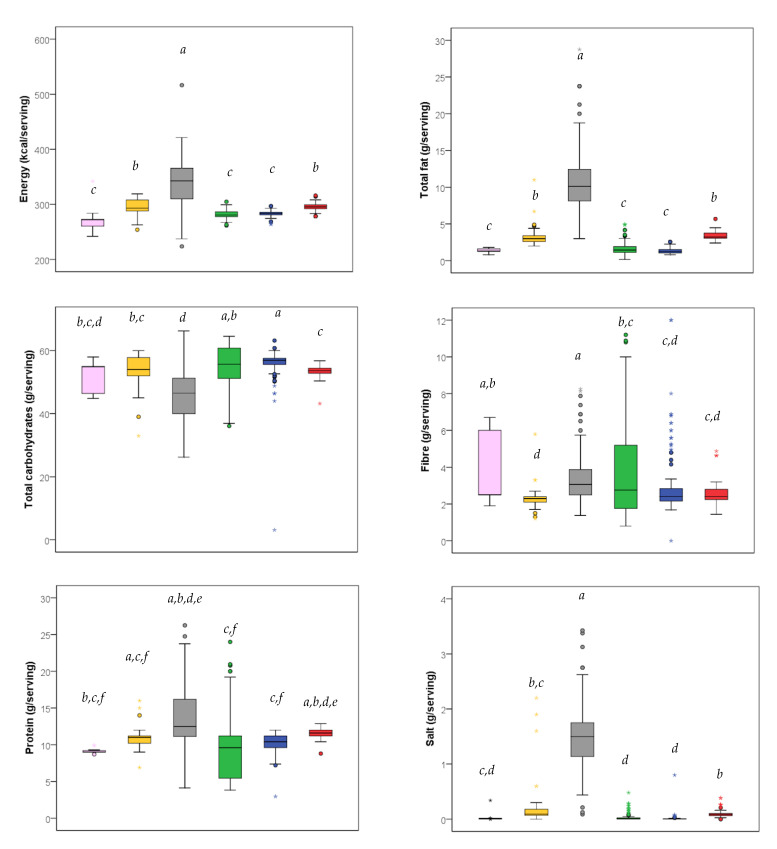
Box plot for energy and nutrition facts per standard serving across categories of pasta. Standard serving was 80 g for all types of dried pasta, 100 g for fresh semolina and egg pasta, and 125 g for fresh stuffed pasta. For each category, different letters indicate significant differences among pasta types (Kruskal–Wallis test for independent samples with multiple pairwise comparisons). 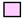 fresh semolina (*n* = 16), 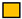 fresh egg (*n* = 45), 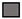 fresh stuffed (*n* = 208), 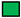 dried special (*n* = 119), 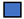 dried semolina (*n* = 157), 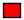 dried egg (*n* = 206).

In order to better investigate the nutrition content of all pasta types, a further evaluation of nutrition facts (energy, nutrients, and salt) per standard serving was performed (Figure 2). This analysis was performed because the different types of pasta can be considered as alternatives as indicated in the Reference Intakes of nutrients and energy for the Italian population [22]. The analysis was carried out without dried stuffed pasta, because only five items were present. Regarding the energy content per standard serving, for fresh stuffed pasta (342 (310–366) kcal/serving) it was significantly higher than for the other pasta types, while no statistical differences were observed among fresh semolina (272 (258–273) kcal/serving), dried semolina (283 (281–286) kcal/serving), and dried special pasta (281 (278–286) kcal/serving), and between fresh egg (293 (288–308) kcal/serving) and dried egg pasta (295 (292–299) kcal/serving). The same trend was observed for total fats. Regarding the carbohydrate content per standard serving, fresh stuffed pasta and dried special pasta showed the greatest variability. In fact, the median carbohydrate content per standard serving of fresh stuffed pasta was significantly lower than other pasta types, except for fresh semolina pasta; moreover, dried semolina pasta had a median content similar to dried special pasta but significantly higher compared to all the other pasta types. Fibre content of dried special pasta had great variability and its median content did not show significant differences from fresh and dried semolina pasta. The median quantity of protein per serving was significantly higher in stuffed pasta compared to the others, except for dried egg pasta. Considering the salt content per standard serving, only fresh stuffed pasta had a median high quantity of salt (1.5 g/serving).

## 4. Discussion

To the best of our knowledge, the present study evaluated, for the first time, the nutritional quality of the different pasta products sold on the Italian market, taking into account the mandatory and some voluntary nutrition information printed on the packaging.

The first intriguing finding is related to the high number of items retrieved on the market. On the one hand, this number confirms Italians as the number one producers and consumers of pasta. It is worth remembering that pasta is indeed a widely common staple food and it is a key product in the Mediterranean dietary pattern [23]—to such an extent that the Italian Food Dietary Guidelines suggests the consumption of one serving of pasta per day (or rice or other cereals) [24]. On the other hand, the several different types of pasta found on the market confirm the great interest of food companies in satisfying the emerging needs and trends of the customers, not only in terms of format but mostly for the many ingredients that can be used for pasta-making.

Data of the nutrition facts evidenced wide differences in terms of energy and nutrients across the two different pasta categories under study, i.e., fresh and dried pasta. Differences were also found among the types of pasta, particularly for stuffed pasta, showing a lower carbohydrate and a higher fat, sugar, and energy content with respect to the other pasta types. Such a difference observed for stuffed pasta is probably mainly due to the filling, of which the weight usually represents half of the total weight. For the same reason, this type of pasta was characterised by the highest median content of salt, with almost all stuffed products (98.6%) having a medium or high salt content. Conversely, the results indicated a negligible amount of salt, the least amount of all pasta types. This is why semolina pasta and other types of pasta are generally prepared at home as well as in the restaurant, canteens, etc. by adding salt in the boiling water; however, the final salt content is likely lower than those reported on the food label of stuffed pasta [25,26].

These results are even more evident by analysing the nutrition declaration per serving size instead of 100 g, mainly because the reference serving for stuffed pasta is generally higher than the ones for dried and fresh semolina and egg pasta [22]. This led to an increased differentiation between the nutritional quality of the different types of pasta, for instance with fresh stuffed pasta providing the highest energy per portion despite no differences were found for 100 g.

Regarding salt, values per serving confirmed the ones obtained per 100 g, highlighting that a portion of stuffed pasta contributes to a high extent to the daily intake of salt, on average equivalent to about 33% of the 5 g/day indicated as the goal by the World Health Organisation (WHO) [27] and over 2 g/serving size for 10% of the stuffed items. It is worth remembering that an excessive consumption of salt in a diet increases blood pressure and consequently the risk of adverse effects on cardiovascular health [28,29]. These results highlight, on the one hand, the importance of nutritional education and increasing knowledge in the population in taking into consideration the serving size, which can deeply influence the nutrient intake; on the other hand, the results suggest that not only food per se, but also the preparation of foods (e.g., adding salt to boiling water), has a key role in the daily intake of nutrients. For example, the high salt content in stuffed pasta suggests that the addition of salt to boiling water should be avoided, even though this is not usually reported on the pack; in some cases, it is even suggested to add salt.

To investigate the nutrition quality of the different categories and types of pasta, we also compared the nutrition facts of products with and without three different declarations, i.e., GF, organic, and NC. This aspect was taken into consideration because consumers’ perception may be influenced by several types of declarations, with the so-called “halo effect” [30]. It has, indeed, been evidenced that consumers perceive foods with claims (e.g., NC) or specific front-of-pack labelling [31], as well as GF products [32] and organic foods [33], healthier than their counterparts. Thus, this misperception may influence food habits, which, in turn, may in some cases lead to an overconsumption [34]. To investigate whether the presence of declaration may affect the nutrition quality of the pasta, we selected and compared the nutrition declaration in an equal number of products with and without each of these declarations on the pack, similarly to what already done in a previous study aimed at comparing the nutritional quality of organic vs. conventional products [20]. This choice was due to the gap of product numbers with and without these declarations currently on the market. The first comparison was made between GF and gluten-containing pasta. Our results suggest that GF products had higher carbohydrates and fat contents and lower fibre, sugar, and protein contents compared to the gluten-containing products. It is worth noting that the use of legume flours/ingredients is increasing in order to enhance the nutritional profile of GF products, resulting in significantly higher fibre and protein contents and a lower amount of carbohydrates compared to conventional semolina pasta [35,36]. However, these types of pasta have not been taken into consideration, as there were no gluten-containing counterparts for the comparison. Our results confirm the findings of an Italian survey considering GF pasta sold on the Italian market, although the authors also found a higher energy, SFA, and salt amount in GF pasta compared to the regular ones [37]. Moreover, our results are partially in agreement with the ones found by a Spanish research considering a total of 53 pasta items, 15 of which were GF [38]. The authors confirmed higher total fats and lower sugars for GF pasta, but they also found higher protein and SFA than gluten-containing pasta. Contrasting results in terms of lipid contents have been found in a UK study focusing on both GF and regular whole grain and white pasta [39]. In fact, UK white GF pasta (111 items) showed a lower content of total fat and SFA than the counterparts (96 items), whereas these data were not confirmed for the whole grain items [39].

Regarding the nutritional profile of products boasting NC, only fibre content was significantly higher than in products without claims. This is plausibly due to the fact the almost all NCs found in these products were related to fibre (i.e., 38 items “source or rich in fibre”), while only 7 items boasted a NC claim related to protein. Intriguingly, a survey conducted on 87 pasta and rice items sold on the Irish market found that 31% of the products considered boasted a NC or a HC and that most of the NC referred to fat (including saturated fats) and carbohydrates, followed by sugars and protein [40]. Overall, data from the present study support the hypothesis that NC should not be considered as marker of the overall quality of food products, as already indicated in previous studies on different types of products [21]. This suggests that more effort should be made in nutrition education to avoid misperceptions, which lead to inappropriate food choices and possibly overconsumption [41].

Finally, we also compared the nutritional quality of organic and conventional pasta. In agreement with studies comparing conventional and organic durum wheat products [42,43,44], our data only showed a significantly lower protein content and a higher fibre amount in organic pasta with respect to the conventional counterpart. Even though no other significant difference between organic and conventional products was found, these variations were not due to the co-existence of other characteristics, such as a different number of wholemeal in the organic vs. the conventional counterparts, as none of the 49 paired items were wholemeal. These results are in line with a previous publication, where it was highlighted that the organic certification cannot be intended as a marker of the general nutritional quality of the products [20].

Our work showed strengths and limitations, mainly attributable to the methodology used for product selection. On the one hand, we analysed for the first time the nutritional composition of a high number of different pasta products retrieved from the major retailers present on the Italian market that have a home-shopping website, thus including the majority of pasta sold in Italy. On the other hand, the exclusion of products sold by local groceries and discounts as well as shops dedicated to special foods, i.e., GF items, might have limited the product analysis. Another limitation of the study concerns the different origin of the nutritional data on the label, which could be based on laboratory analysis or calculation from the ingredients used or generally established and accepted data, creating a putative bias in data origin. Moreover, the high variability of filling characteristics found among stuffed pasta items made tricky the intra- and inter-type comparisons of the nutritional quality. Finally, the comparison of the nutritional quality between the pairs of products with and without declarations could be considered a limitation. However, including all the 756 items in the analysis would have affected the findings because of the large difference in the number of products of the same type, with and without declarations such as GF, organic, or NC. Conversely, the comparison of items of the same brand was a way to avoid that the brand name can act as a possible cause of bias.

## 5. Conclusions

To the best of our knowledge, this is the first study which comprehensively analysed the nutritional composition of a wide range of fresh and dried pasta products sold in the Italian market. Data showed that pasta types currently on the market are very different in terms of nutrition profile, and not really comparable. Particularly stuffed pasta was characterised by a high salt content, representing a large proportion of the maximum of 5 g/day indicated by the WHO. This last aspect particularly highlights the need to clarify as much as possible the nutrition facts of product to the consumer, as pasta is usually eaten by adding sauces and/or toppings which might further increase the energy, macronutrient, and salt intake. Linked to this, we also advised that salt, mainly the discretionary one, should be carefully reduced or avoided in cooking this type of pasta. It is, indeed, particularly crucial to increase consumer awareness about the choice of both adequate pasta type and dressing and their contribution to the nutritional quality of the entire dishes.

Overall, findings from the present study are particularly of interest and should be taken into account in dietary recommendations, which currently provide only information regarding the serving size of the different type of pasta, but not about their frequency of consumption. Stuffed pasta probably should not be regularly consumed as an alternative to semolina pasta. Thus, the awareness of the consumers about the nutrition profile of the different types of pasta could be the topic of targeted nutrition education interventions aimed to improve their knowledge and, in turn, their food habits. Finally, with this study focusing on pasta products, we confirm that organic or other declarations, i.e., NC, cannot be an overall marker of the nutritional quality of the product and, thus, this topic should also be the object of future nutrition education targeted to consumers.

## Figures and Tables

**Table 1:** Energy and nutritional composition across categories of pasta.

	Fresh Pasta				Dried Pasta				
	All (*n =* 269)	Semolina (*n =* 16)	Egg (*n =* 45)	Stuffed (*n =* 208)	All (*n =* 487)	Semolina (*n =* 157)	Egg (*n =* 206)	Stuffed (*n =* 5)	Special (*n =* 119)
Energy (kcal/100 g)	280 (253–295)	272 (261–273) ^b^	293 (288-308) ^a^	274 (248–293) ^b^	359 (352–368)	354 (351–357) ^b^	369 (365–374) ^a^	394 (393–403) ^a^	351 (347–358) ^b^
Total Fat (g/100 g)	7.3 (4.7–9.2)	1.3 (1.2–1.6)	3.0 (2.6–3.4) ^b^	8.1 (6.5–10.0) ^a^	2.5 (1.5–4.0)	1.5 (1.3–1.9) ^c^	4.0 (3.8–4.7) ^b^	12.5 (12.2–13.0) ^a^	1.8 (1.4–2.4) ^c^
SFA (g/100 g)	2.7 (1.6–3.8)	0.3 (0.3–0.3) ^b^	1.0 (0.7–1.3) ^b^	3.1 (2.4–4.1) ^a^	0.7 (0.3–1.2)	0.4 (0.3–0.5) ^b^	1.3 (1.2–1.5) ^a^	3.0 (2.7–3.8) ^a^	0.4 (0.3–0.5) ^b^
Total Carb (g/100 g)	39.9 (33.6–47.0)	54.9 (46.4–55.0) ^a^	54.0 (52.0–57.8) ^a^	37.2 (32.0–41.0) ^b^	68.0 (66.0–71.5)	71.0 (69.5–72.0) ^a^	67.0 (66.0–68.0) ^b^	51.7 (51.0–52.9) ^b^	69.6 (64.0–76.0) ^a^
Sugars (g/100 g)	2.4 (1.4–4.0)	1.5 (1.5–1.6) ^b^	1.3 (1.1–1.5) ^b^	3.1 (1.7–4.6) ^a^	2.8 (2.2–3.2)	3.2 (2.9–3.5) ^a^	2.8 (2.4–3.0) ^b^	3.2 (2.0–3.3) ^ab^	1.4 (0.6–2.6) ^c^
Fibre (g/100 g) ^#^	2.4 (2.0–3.1)	2.5 (2.5–6.0) ^a^	2.3 (2.1–2.4) ^b^	2.5 (2.0–3.1) ^b^	3.0 (2.8–3.8)	3.0 (2.7–3.6)	3.0 (2.8–3.5)	5.0 (3.3–7.5)	3.5 (2.2–6.5)
Protein (g/100 g)	10.0 (9.0–12.0)	9.0 (9.0–9.2) ^b^	11.0 (10.2–11.2) ^a^	10.0 (8.9–13.0) ^a^	14.0 (12.0–15.0)	13.0 (12.0–14.0) ^b^	14.5 (14.0–15.0) ^a^	15.3 (15.0–15.5) ^a^	12.0 (6.8–14.0) ^b^
Salt (g/100 g)	1.1 (0.6–1.4)	0.0 (0.0–0.0) ^b^	0.1 (0.1–0.2) ^b^	1.2 (0.9–1.4) ^a^	0.0 (0.0–0.1)	0.0 (0.0–0.0) ^b^	0.1 (0.1–0.1) ^a^	1.0 (1.0–1.0) ^a^	0.0 (0.0–0.0) ^b^

Values are expressed as median (25th–75th percentile). SFA: saturated fatty acid; Carb: carbohydrate. For each category, different superscript lowercase letters in the same row indicate significant differences among types (Kruskal–Wallis test for independent samples with multiple pairwise comparisons). ^#^ Number of items reporting fibre: Fresh pasta (All *n* = 198; Semolina *n* = 15; Egg *n* = 34; Stuffed *n* = 149), Dried pasta (All *n* = 449; Semolina *n* = 135; Egg *n* = 193; Stuffed *n* = 5; Special *n* = 116).

**Table 2:** Energy and nutrition facts in products with and without specific declarations, on selected pairs of products (as reported in Materials and Methods section).

	Organic		GF		NC	
	Yes (*n* = 49)	No (*n* = 49)	Yes (*n* = 90)	No (*n* = 90)	Yes (*n* = 45)	No (*n* = 45)
Energy (kcal/100 g)	357 (351–365)	359 (354–366)	349 (283–358)	353 (291–356)	350 (346–361)	349 (346–359)
Fat (g/100 g)	2.0 (1.4–3.8)	2.0 (1.4–3.9)	2.3 (1.5–7.1)	1.6 (1.4–6.0) *	2.1 (1.5–3.3)	2.4 (1.7–3.3)
SFA (g/100 g)	0.6 (0.3–1.0)	0.5 (0.4–1.3)	0.7 (0.3–2.6)	0.4 (0.3–2.4)	0.4 (0.3–1.0)	0.4 (0.3–0.9)
Carbohydrates (g/100 g)	70.6 (67.5–72.0)	69.0 (67.0–70.8)	73.7 (42.0–77.2)	70.1 (42.0–71.5) *	66.5 (64.6–68.0)	66.0 (63.0–69.4)
Sugars (g/100 g)	2.6 (2.4–3.5)	2.9 (2.6–3.5)	0.7 (0.4–1.3)	3.2 (2.9–3.7) *	2.7 (2.3–3.2)	2.5 (1.5–3.0)
Fibre (g/100 g) ^#^	3.0 (2.8–3.4)	2.8 (2.7–3.0) *	2.3 (2.0–3.1)	2.7 (2.5–3.0) *	6.1 (3.4–7.0)	4.0 (2.8–6.5) *
Protein (g/100 g)	12.0 (11.0–14.0)	13.7 (13.0–14.6) *	7.2 (6.5–8.5)	12.6 (11.2–13.5) *	13.0 (11.1–14.2)	13.0 (10.5–14.0)
Salt (g/100 g)	0.0 (0.0–0.1)	0.0 (0.0–0.1)	0.0 (0.0–0.8)	0.0 (0.0–1.0)	0.0 (0.0–0.1)	0.0 (0.0–0.1)

Values are expressed as median (25th–75th percentile). SFA: saturated fatty acid; GF: gluten free; NC: nutrition claim. For each category, asterisks indicate significant differences between groups (Mann-Whitney non-parametric test for two independent samples), *p* < 0.05. ^#^ Number of items reporting fibre: organic = 45/43; GF = 82/74; NC = 45/43 (yes/no).

## Data Availability

Not applicable.
